# Circadian variations in the liver metabolites of medaka (*Oryzias latipes*)

**DOI:** 10.1038/srep20916

**Published:** 2016-02-10

**Authors:** Koichi Fujisawa, Taro Takami, Yoshitaka Kimoto, Toshihiko Matsumoto, Naoki Yamamoto, Shuji Terai, Isao Sakaida

**Affiliations:** 1 Center for Regenerative Medicine, Yamaguchi University School of Medicine, Minami Kogushi 1-1-1, Ube Yamaguchi 755-8505, Japan; 2 Department of Gastroenterology and Hepatology, Yamaguchi University Graduate School of Medicine, Minami Kogushi 1-1-1, Ube Yamaguchi 755-8505, Japan; 3 Division of Gastroenterology and Hepatology, Graduate School of Medical and Dental Sciences, Niigata University, 1–757 Asahimachidori, Chuo-Ku, Niigata 951–8510, Japan

## Abstract

Circadian rhythms are biological rhythms with a period of around 24 hours. In this study, we compared the metabolome of the liver of medaka during the day and night. To comprehensively analyze the circadian variations in the levels of metabolites in the liver, livers were isolated from Zeitgeber time (ZT)4 and ZT16, and the variations in metabolite levels were evaluated. Inosinemonophosphate (IMP) and uridinemonophosphate (UMP) were found to be increased at night, indicating that nucleotide synthesis is most active during the night. Furthermore, the levels of metabolites of the tricarboxylic acid cycle were also reduced at night. In addition, the levels of many amino acids were reduced during the night, suggesting that the amino acids had been degraded. Moreover, the citrulline/ornithine ratio, which is related to arginine consumption, was lower during the day than at night. This pattern suggests that the urea cycle is activated during the day, whereas large amounts of nitric oxide and citrulline may be produced from arginine via nitric oxide synthase during the night. The results of this metabolomic analysis may be useful in future fundamental research to provide insight into chronobiology as well as applied research on drug evaluations using medaka as a model species.

Many organisms display functions with a biological rhythm over a period of approximately 24 hours, which is known as the circadian rhythm; these physiological function include sleeping and waking, hormone secretion, and the regulation of blood pressure and temperature. The core genes encoding the proteins that form the molecular mechanism underlying the circadian rhythm followed in these functions are collectively known as the clock genes, which include *Period* (*Per*), *Clock*, and *Bmal1*, and their transcription and translation forms a feedback loop, which is an integral part of the circadian clock^1^. Recent research progress in molecular biology has helped to provide new insight into the function and expression patterns of clock genes. For example, it has become clear that clock genes are present in most peripheral tissues, from the heart, blood vessels, liver, and kidney, to the skin and mucous membranes^2^. Furthermore, the relationship between circadian rhythms and aging, osteoporosis, oncogenesis, and lifestyle diseases such as hypertension, hyperlipidemia, and diabetes is also actively investigated^3^.

Small fish such as medaka (*Oryzias latipes*) and zebrafish have emerged as new useful vertebrate model organisms. One of their main advantages is their smaller size, requiring less space and making them inexpensive to rear compared to typical rodent models such as mice. Other advantages of using small fish as vertebrate models include the well-established methods for creating transgenic and knockout animals since the completion of the genome project. Furthermore, the rapid development and high fecundity enable detailed analyses of the development processes of small fish, thereby facilitating large-scale screening^4^. Specifically, medaka has the advantages of small size, established models produced from inbred lines, rapid development and high fecundity, and omnivorousness; further medaka also shows sugar and lipid metabolic profiles similar to those of mammals^5^. Furthermore, in contrast to mice, which are nocturnal, zebrafish and medaka are diurnal species, and because a large number of individuals can be reared under the same conditions within identical tanks, these species are well suited for research on circadian rhythms. Despite these advantages, most of the research into the biological rhythms produced by clock genes has focused on mammals, and most of the studies on fish have used zebrafish as a model. Li *et al.* reported usefulness of a spatial gene expression atlas to investigate the expression of circadian genes in various tissues and cell types^6^. Recently, they also developed a computational method to integrate both circadian gene expression and metabolic network metabolomics analysis, and reported that the level of IMP, an intermediate metabolite in de novo purine synthesis, showed significant circadian oscillation in larval zebrafish^7^. Indeed, there has been only one report on the circadian rhythms of medaka published to date^8^, which involves a quantitative reverse transcription-polymerase chain reaction (RT-PCR) analysis of the expression of clock genes in the eyes, brain, fins, and heart of adult medaka over a 24-hour period in both light and dark (LD) conditions. However, this previous study neglected the liver, which is the most important metabolism-governing organ. In addition, there has been no metabolomic analysis of circadian rhythms using medaka to date. Thus, we analyzed the circadian rhythm in the livers of adult medaka using a metabolomics approach.

## Materials and Methods

### Animals

Fish of the medaka line, Cab *O. latipes,* were used in this study; all fish were approximately 6 months old. Medaka fish were reared using tap water with aeration at 25 °C. The fish were fed twice daily at 8:00 and 20:00, and the water was changed twice a week. This study was carried out according to Yamaguchi University’s guidelines for animal use. All experimental protocol were approved by the Committee on the Ethics of Animal Experiments of the University of Yamaguchi.

### Twenty-four hour behavior monitoring

The Chronobiology Kit (Stanford Software Systems) was used to observe the amount and periodicity of the movement of 5 medaka fish in a single tank. Observations were made over 10 days, and the results were displayed as an actogram. The LD conditions were established with a light period from 08:00 to 20:00, and a dark period from 20:00 to 08:00, such that L:D = 12:12 (h).

### RT-PCR

The total RNA was isolated from six fish each time using Isogen (Life Technology) according to the manufacturer’s instructions. For cDNA synthesis, Taqman reverse transcription reagents (Roche Diagnostics, Indianapolis, IN, USA) were used as described in the manufacturer’s manual. Variations in the expression of the clock genes *per1*, *bmal1,* and the control *18S rRNA* gene were analyzed using a Step One Plus real-time PCR system (Life Technologies) with SYBR green. For the RT-PCR analysis, primers were chosen for their dissociation curves, lack of non-specific amplification, and relatively good amplification efficiency. The base sequences for the utilized primers are as follows:

*per1* Forward 5′-TACCACCAGTGGAGTGTGGA-3′, Reverse 5′-AGGTGTCCGTGTTTTTCAGG-3′; *bmal1* Forward 5′-CCATGTCCCGCAAGTTGGAC-3′, Reverse 5′-GCAATGTCCTTGGGATGCAG-3′; *18S rRNA* Forward 5′- AAGCAGGCCCGGTCGCCTGAATACC-3′, Reverse 5′- AATCGCTCCACCAACTAAGAACGGCCATGC-3′.

The data shown is a representative triplicate experiment.

### Measurement of metabolites

Approximately 50 mg of frozen tissue from five fish (n = 3) was plunged into 1,500 μL of 50% acetonitrile/Milli-Q water containing internal standards (Solution ID: 304–1002, Human Metabolome Technologies, Inc., Tsuruoka, Japan) at 0 °C in order to inactivate enzymes. The tissue was homogenized thrice at 1,500 rpm for 120 sec using a tissue homogenizer (Micro Smash MS100R, Tomy Digital Biology Co., Ltd., Tokyo, Japan) and then the homogenate was centrifuged at 2,300 × g and 4 °C for 5 min. Subsequently, 800 μL of upper aqueous layer was centrifugally filtered through a Millipore 5-kDa cutoff filter at 9,100 × g and 4 °C for 120 min to remove proteins. The filtrate was centrifugally concentrated and re-suspended in 50 μL of Milli-Q water for CE-MS analysis. CE-TOFMS was carried out using an Agilent CE Capillary Electrophoresis System equipped with an Agilent 6210 Time of Flight mass spectrometer, Agilent 1100 isocratic HPLC pump, Agilent G1603A CE-MS adapter kit, and Agilent G1607A CE-ESI-MS sprayer kit (Agilent Technologies, Waldbronn, Germany). The systems were controlled by Agilent G2201AA ChemStation software version B.03.01 for CE (Agilent Technologies, Waldbronn, Germany). The metabolites were analyzed by using a fused silica capillary (50 μm i.d. × 80 cm total length), with commercial electrophoresis buffer (Solution ID: H3301-1001 for cation analysis and H3302-1021 for anion analysis, Human Metabolome Technologies) as the electrolyte. The sample was injected at a pressure of 50 mbar for 10 sec (approximately 10 nL) in cation analysis and 25 sec (approximately 25 nL) in anion analysis. The spectrometer was scanned from m/z 50 to 1,000. Peaks were extracted using automatic integration software MasterHands (Keio University, Tsuruoka, Japan) in order to obtain peak information including m/z, migration time for CE-TOFMS measurement (MT) and peak area. Signal peaks corresponding to isotopomers, adduct ions, and other product ions of known metabolites were excluded, and remaining peaks were annotated with putative metabolites from the HMT metabolite database based on their MTs and m/z values determined by TOFMS. The tolerance range for the peak annotation was configured at ±0.5 min for MT and ±10 ppm for m/z. In addition, peak areas were normalized against those of the internal standards and then the resultant relative area values were further normalized by sample amount. To identify the affected metabolic pathways, a proof-of-knowledge based Ingenuity Pathway Systems (IPA, Redwood City, CA, USA) analysis was performed.

### Statistical analysis

The results were analyzed by either the Student’s t-test or two-way analysis of variance, and the data are presented as mean ± standard deviation with significance level established at p < 0.05.

## Results

### Evaluation of the circadian rhythm in medaka

**Figure 1:**
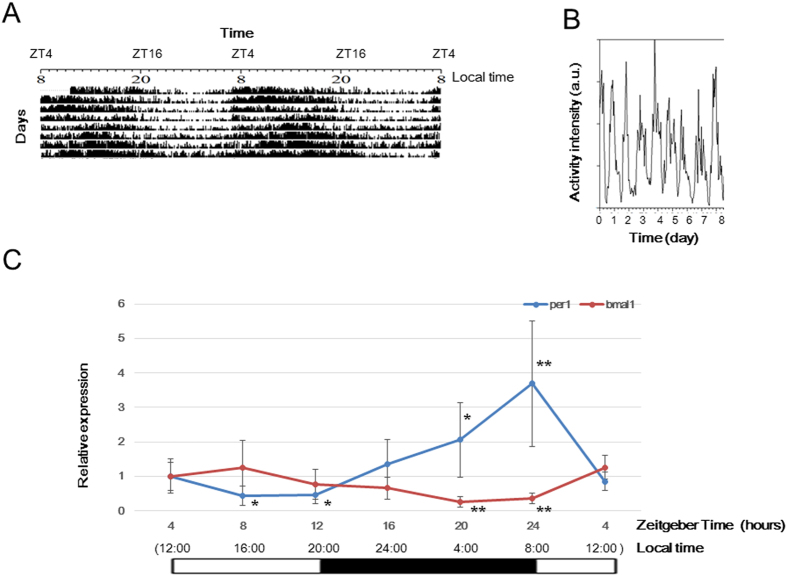
Locomotor activity and 24-hour variations in clock gene expression in the medaka liver. (**A**) Representative double-plotted actograms of adult medaka. Each tank contained 5 medaka, with light-dark conditions consisting of a light period (08:00–20:00) and a dark period (20:00–8:00) for an L:D ratio of 12:12 h. (**B**) Overall change in daily activity of adult medaka by time series. A series of points representing the average of data is plotted. Step size is 60 mins. (**C**) Variations in *per1* and *bmal1* expression levels in the adult medaka liver under light and dark conditions (12:12 LD cycle). Levels are expression relative to *18S rRNA* expression. The black and white bars in the upper panel show the light and dark periods, respectively. The horizontal axis is time, and the vertical axis shows the values relative to ZT4. All P-values were evaluated using the Student’s t-test. *P < 0.05, **P < 0.01

Upon observing the amount of movement of medaka during a 24-hour period, the actogram plot showed that the medaka were more active during the day, and that their movement reduced markedly during the night (Fig. 1A,B). In addition, to investigate the diurnal variation in the expression of various genes in the medaka liver, RNA was isolated from livers harvested every 4 hours (at Zeitgeber time [ZT] 4, ZT 8, ZT 12, ZT 16, ZT 20, and ZT 24) and subjected to real-time RT-PCR analysis. Variation in the expression of the clock genes *per1* and *bmal1* levels was investigated using *18S rRNA* as a control gene. For *per1*, a marked increase in expression was found during the early morning (Fig. 1C), whereas the expression of *bmal1* was found to decline during the early morning (Fig. 1C).

### Metabolomic analysis between day and night

**Figure 2:**
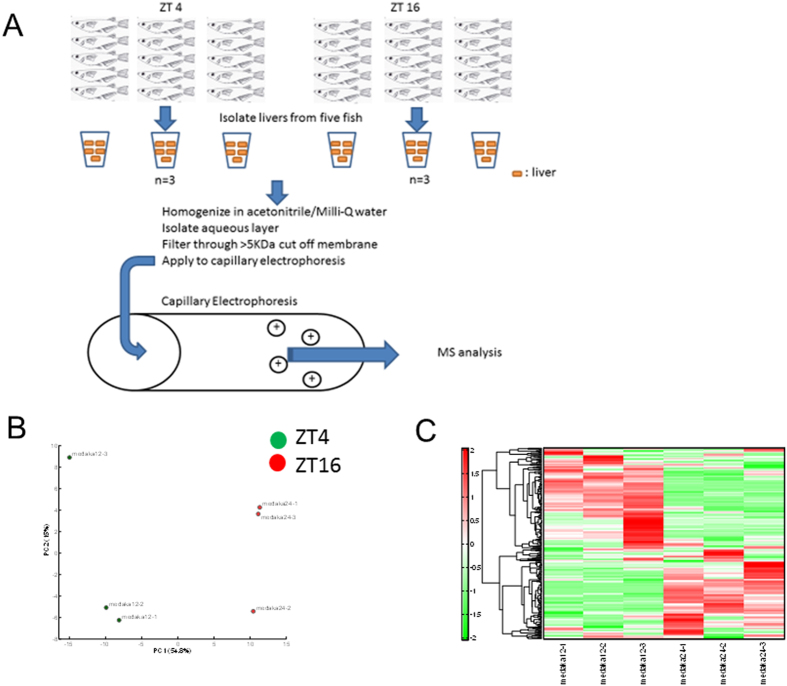
Principal component analysis (PCA) of the medaka liver metabolites during the day and at night. (**A**) schematic diagram of preparation of samples and CETOFMS (**B**) Principal component analysis-normalized metabolic data obtained from the medaka liver at ZT4 and ZT16. Percentage values indicated on the axes represent the contribution rate of the first (PC1) and second (PC2) principal components to the total amount of variation. (**C**) Heat map of the hierarchical cluster analysis. The columns are the medaka liver metabolome profiles at ZT4 and ZT16. The rows show the normalized levels of each metabolite. The dendrogram for each heatmap shows the relatedness of the normalized metabolite level patterns.

To more comprehensively evaluate the circadian variations in the metabolite levels of the liver, the livers were isolated from the medaka at ZT4 and ZT16, and variations in metabolite levels were evaluated using CE-TOFMS analysis (Fig. 2A). In the principal components analysis, the day and night groups were clearly differentiated along the PC1 axis, accounting for the majority (54.8%) of the variation between the two groups (Fig. 2B). Furthermore, hierarchical clustering showed that the relative abundance of metabolites was reversed between the day and night groups (Fig. 2C).

#### Purine and pyrimidine metabolism

**Figure 3:**
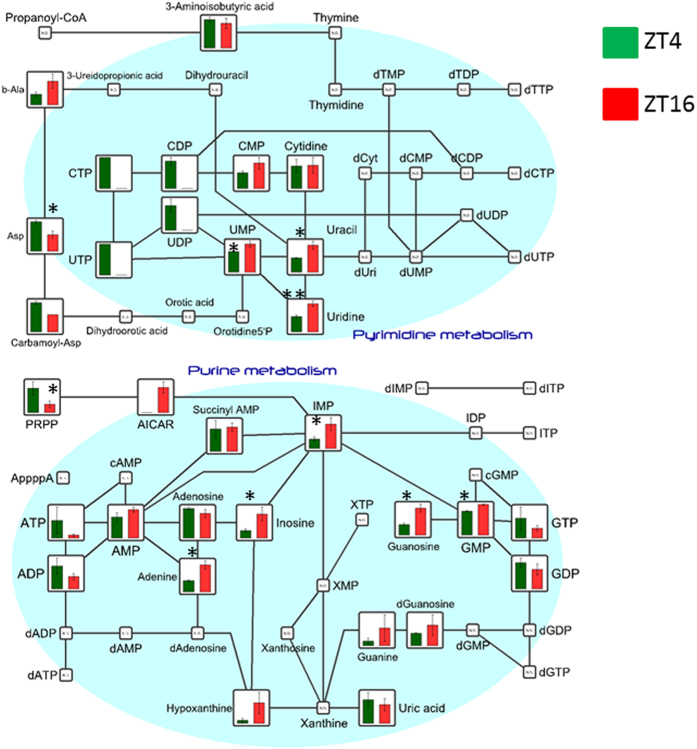
Comparison of metabolites involved in pyrimidine and purine metabolism. The metabolites are superimposed on a metabolite pathway map involving the metabolism of pyrimidines and purines. All P-values were evaluated using the Student’s t-test. *P < 0.05, **P < 0.01

Nucleotide metabolism is associated with cell proliferation and urate synthesis. Variation in nucleotide synthesis in the liver is particularly important, as the liver provides the nucleotides necessary to maintain the functions of other organs. Analysis of metabolites related to purine and pyrimidine metabolism revealed an increase in monophosphates at night, including 3′-cytidine monophosphate (CMP; day-to-night ratio = 1.8), uridine monophosphate (UMP; ratio = 1.3), guanosine monophosphate (GMP; ratio = 1.3), and inosine monophosphate (IMP; ratio = 2.6) (Fig. 3). Significant variations were also found in inosine (ratio = 3.2), guanosine (ratio = 2.6), uracil (ratio = 1.9), adenine (ratio = 2.4), uridine (ratio = 1.9), phosphoribosyl pyrophosphate (PRPP; ratio = 0.3), and UDP-glucose (ratio = 2.8) (Fig. 3). Although a tendency towards reduced ATP and GTP levels at night was observed, the difference was not statistically significant.

#### The glycolytic pathway and tricarboxylic acid (TCA) cycle

**Figure 4:**
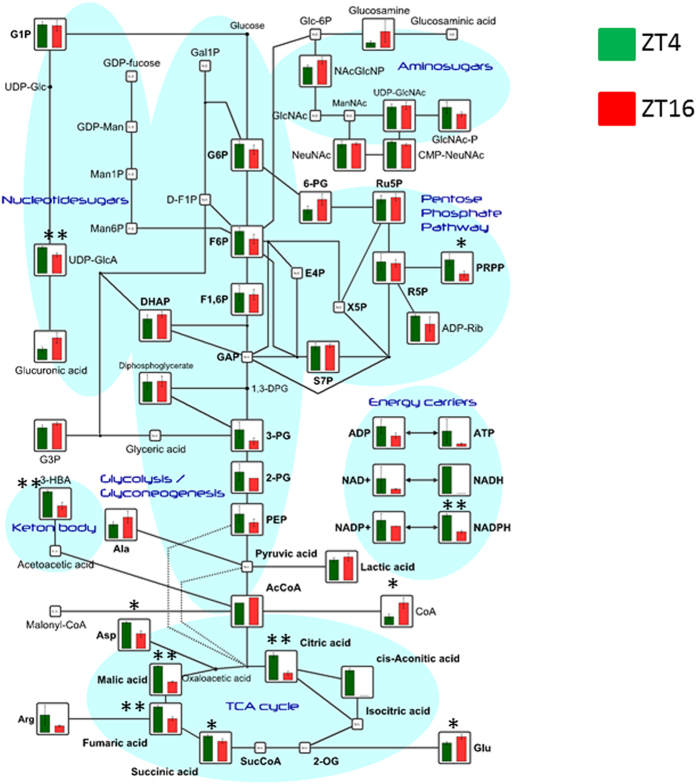
Comparison of the metabolites of central carbon metabolism. The metabolites are superimposed on metabolite pathway maps of glycolysis, the pentose phosphate pathway, and the tricarboxylic acid (TCA) cycle. All P-values were evaluated using the Student’s t-test. *P < 0.05, **P < 0.01

To compare energy metabolism between day and night in the livers of medaka, variations in metabolites related to the glycolytic pathway and TCA cycles were evaluated. Of the metabolites of the TCA cycle, reduction at night was observed for succinic acid (ratio = 0.8), fumaric acid (ratio = 0.5), malic acid (ratio = 0.4), and citric acid (ratio = 0.3) (Fig. 4). Despite a tendency towards reduced levels for many metabolites of the glycolytic pathway at night, no significant differences were found.

#### Amino acids

Most amino acids reduced during the night (Table 1), including isoleucine (ratio = 0.6), threonine (ratio = 0.6), His (ratio = 0.6), lysine (ratio = 0.5), and methionine (ratio = 0.2). Increases at night were observed for sarcosine (ratio = 5.1) and glycine (ratio = 2.2).

#### Lipid metabolism

**Figure 5:**
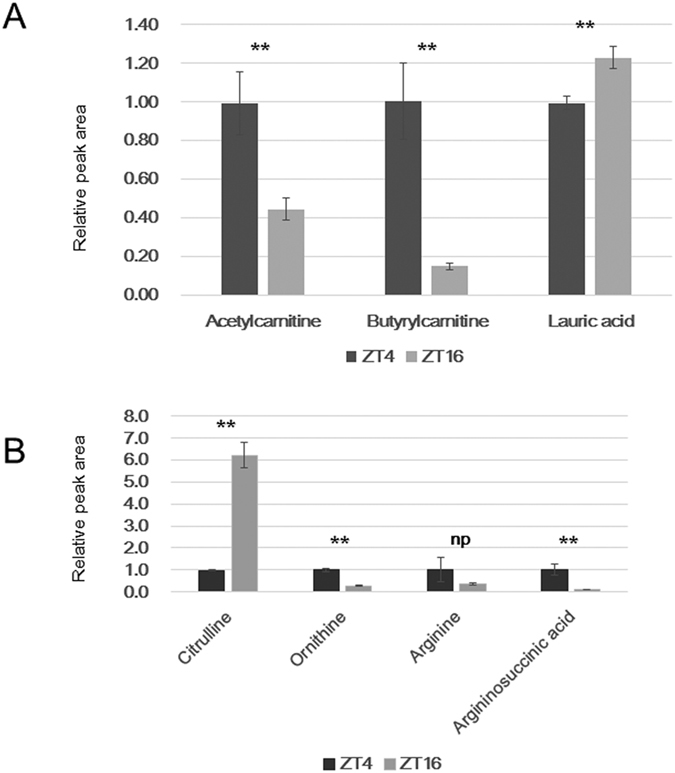
Relative changes in the metabolites of lipid metabolism and the urea cycle between day and night. (**A**) Relative changes in the metabolites of lipid metabolism. (**B**) Relative changes in ornithine, citrulline, arginine and arginosuccinate. *P < 0.05, **P < 0.01.

For the lipid analysis, we were only able to evaluate hexanoic acid, lauric acid, and octanoic acid in the CE-TOFMS analysis. Lauric acid was found to be significantly elevated in the night group (Fig. 5A). Although there was a tendency towards increased hexanoic acid and octanoic acid levels at night, the difference was not statistically significant. In addition, a reduction in acetylcarnitine and butyrylcarnitine was found in the night group, suggesting an increase in fatty acid synthesis and a reduction in fatty acid β-oxidation at night.

#### Urea cycle

The urea cycle is a metabolic cycle, in which urea is produced from ammonia in the mitochondria and cytoplasm of hepatic cells. Among the metabolites of the urea cycle, ornithine levels were increased during the day, whereas citrulline levels were increased during the night (Fig. 5B).

### IPA analysis

**Figure 6:**
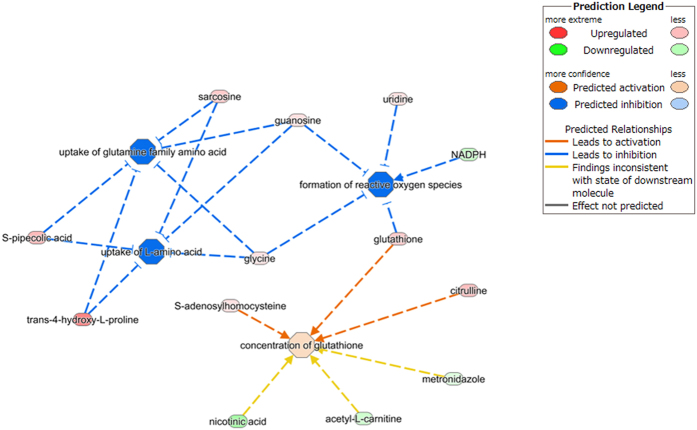
Network of formation of reactive oxygen species, concentration of glutathione, uptake of glutamine family amino acid, and uptake of L-amino acid identified by IPA. The network indicates how the functions “formation of reactive oxygen species”, “concentration of glutathione”, “uptake of glutamine family amino acid” and “uptake of L-amino acid” are predicted by virtue of the metabolites. Color is used to indicate both the direction (red, upregulation; green, downregulation) and the magnitude (color intensity) of the expression change of the target metabolites.

The results of the canonical pathway analysis by IPA are shown in Table 2. The adenine and adenosine, guanine and guanosine, and pyrimidine ribonucleotide salvage pathways, and the *de novo* biosynthesis of purine nucleotides were identified to be significantly modulated during the day and night. Furthermore, the citrulline-nitric oxide cycle, a superpathway of citrulline metabolism, and the urea cycle were determined to be canonical pathways with significant differences between the night and day groups. We also analyzed disease and function network by IPA, and found that formation of reactive oxygen species (*p*-value: 3.54E-02, activation Z-score: −2.213), uptake of glutamine family amino acid (*p*-value: 2.87E-03, activation Z-score: −2.236), and uptake of L-amino acid (*p*-value: 1.73E-02, activation Z-score: −2.219) were predicted to be decreased, and concentration of glutathione (*p*-value: 9.24E-03, activation Z-score: 0.298) was predicted to be increased (Fig. 6).

## Discussion

A coupling of metabolism and the circadian rhythm has been established in various organisms from humans to yeast. Most of the early research on circadian rhythms was focused on mammals, although research with fish species has increased in recent years, owing to their simplicity, ease of rearing, and the breadth of molecular knowledge that has accumulated in the last few decades. However, there are some demerits of small fish such as differences in liver structure, shortage of useful antibodies and differences in maintenance system of body temperature. Of the small fish, most research has been conducted with the zebrafish, including analyses of the circadian rhythm during development^9^. Recently, metabolomics analysis of adult zebrafish were reported^6^,^7^. In contrast, there has been only one report on circadian rhythms in adult medaka, which addressed the rhythms in locomotor activity rhythms in relation to the expression of the clock genes *per1* and *bmal1*^8^. However, the expression of *per1* and *bmal1* in the liver was not evaluated in this previous study. In the present study, we analyzed gene expression every 4 hours, and found that *per1* levels increased in the early morning, whereas *bmal1* showed the opposite pattern. Although we expected that *per1* level increase and *bmal1* level decrease just after day time starts, the expressional pattern of these genes shifted relatively earlier. But this result matches the patterns of expression previously reported in the heart and fin^8^. It is known that PER1 plays an important role in biological clock, its expression may have significant effects on the cell cycle and interacts with ATM and Chk2, checkpoint proteins^10^. BMAL1, a master regulator of circadian rhythm, also plays important roles in the regulation of lipogenesis. The rhythm made by these circadian related genes caused metabolomics changes such as purine and pyrimidine metabolism, lipid metabolism and amino acids metabolism.

Most of the studies conducted thus far on metabolomics in relation to circadian have been in mice^11^, or from human samples such as blood and saliva^12^. Since mice are nocturnal mice, data from the diurnal medaka may be more useful with respect to interpretations for humans, particularly regarding metabolomic analysis of the liver. Consequently, we analyzed variations in the liver metabolites in medaka during the day and night, and investigated each metabolic pathway in detail.

Daily variations in nucleotide metabolism are particularly important, since the liver supplies the nucleotides that act as the raw materials for forming the nucleic acids required for the function of other organs. In mice, nucleotides are supplied from the liver to all other organs depending on the liver-specific circadian rhythm. Accordingly, variations in enzyme activity due to the circadian rhythm of clock gene expression in the liver can cause the side effects of anti-cancer drugs (such as 5-FU) to vary widely over the course of a day^13^. The metabolic pathways of nucleotides consist of the *de novo* pathway and the salvage pathway. Purine nucleotides are produced using *de novo* synthesis, whereby d-ribose-5-phosphate supplied by the hexose monophosphate shunt is pyrophosphorylated on the 1′-OH group to become 5 PRPP, and is converted into the final product, IMP. AMP and GMP can then be produced from IMP. In addition, the final product in the *de novo* synthesis pathway of pyrimidine nucleotides is UMP. In the present study, IMP and UMP were found to increase significantly at night, which suggests that nucleotide synthesis is most active during the night. IPA analysis further confirmed significant differences in the activation of nucleotide salvage and biosynthesis pathways during the day and night. Interestingly, it is reported that de novo purine synthesis in the zebrafish mediates circadian control of cell cycle, a mechanism that is likely conserved in mammals^7^. In this report three gene homologs of a key enzyme in this pathway, impdh, show circadian oscillations in different tissues and have distinct molecular functions. In particular, one of the homologs, impdh2, contributes to the daily rhythm of S phase in the cell cycle. A strong relationship between nucleotide metabolic pathways and response to cancer chemotherapy has been identified^14^. From the data of nucleotide, administration of anti-cancer drugs such as nucleotide analogues in night time is less effective for normal tissue (reduced side-effect), but more effective for tumors in which circadian rhythms tend to be disrupted. Therefore, further considering of nucleotide metabolism may help to develop new cancer chemotherapies such as chronotherapy.

Of the many functions of the liver, carbohydrate energy metabolism is one of the most important ones. A previous report demonstrated that the activity of enzymes related to carbohydrate energy metabolism was coordinated with the circadian rhythm^15^. In the present study, a significant nighttime decrease in the metabolites of the TCA cycle, including malate and fumarate, was found, and the metabolites of the glycolytic pathway also tended to decrease, although the difference was not statistically significant. These variations may be related to gluconeogenesis during feeding and fasting. Indeed, aconitase 2, which catalyzes the conversion of citrate into isocitrate in the TCA cycle, is reported to show rhythmic expression^16^. More recently, a study was reported that attracted great attention, which showed that the clock gene feedback loop gives rise to the NAD+ biosynthesis cycle, and that ATP production and mitochondrial respiration are regulated via modulation of mitochondrial protein acetylation by the NAD+ dependent deacetylase SIRT3^17^.

Circadian rhythms in the blood amino acid levels are also well known, with a daily variation of around 30%. In humans, blood amino acid levels are elevated in the afternoon and are reduced during the early morning, and the plasma levels of the amino acid substrates of protein synthesis are elevated during the waking phase^18^. In the present study, the levels of many amino acids were found to be reduced during the night, except for cysteine, glutamate and glycine, which were found to increase at night. These results generally suggest that amino acid degradation occurs at night, which may serve to increase blood glucose levels while fasting. We analyzed disease and function network by IPA, and found that uptake of glutamine family amino acid and uptake of L-amino acid were predicted to be decreased (Fig. 6). Although most of amino acids decreased, 3 amino acids (Cysteine, Glycine and Glutamate) increased. It is interesting that all increased amino acids, Cysteine, Glycine and Glutamate, are component of glutathione (GSH). GSH is known to be a central player in the antioxidant defense network and redox-sensitive signaling. The changes in GSH level were observed in different mammalian organs, along with daily changes in the levels of GSH-biosynthetic gene products^19^. Branched chain amino acid (BCAA) is known as an effective drug for liver disease, and administration of BCAA in night time might be more effective from the point of view of chronotherapy.

In recent years, more attention has been paid to the relationship between lipid metabolism and circadian rhythms with respect to their potential association with lifestyle-related diseases. In the present study, it was only possible to evaluate hexanoic acid, lauric acid, and octanoic acid in the CE-TOFMS analysis. There was a tendency towards elevated hexanoic acid and octanoic acid levels at night, although no significant difference was found. However, a significant increase in lauric acid was found for the night group, suggesting an increase in fatty acid synthesis. In addition, a reduction in acetylcarnitine and butyrylcarnitine was found in the night group, suggesting a reduction in fatty acid β-oxidation at night. *bmal1* is thought to be related to lipogenesis and lipolysis. The expression of *bmal1* dropped in early morning in medaka liver. Higher activity of lipolysis in ZT4 may be related to *bmal1* expression. In mice, it has been suggested that the clock gene levels become elevated during the inactive period, and the subsequent elevation of PPAR expression leads to β-oxidation^20^. Even more recently, interest has grown around the role of sirtuin and lipid metabolism with respect to circadian rhythms. In the liver, fasting causes activation and elevation of SIRT1 protein levels, inhibition of the glycolytic pathway, and regulation of fatty acid oxidation by PGC1 and PPAR^21^. The phenomenon of elevated lipid anabolism during the inactive period, as found in the present study, is opposite to findings of the inactive period in other organisms, in which elevated fatty acid catabolism has been observed. Therefore, this relationship between fatty acid levels and circadian rhythms within and among species requires further detailed analysis.

The urea cycle is important for the function of hepatic cells, and ornithine and citrulline levels are known to vary periodically. In addition, it is reported that carbamoyl-phosphate synthetase 1 (CPS1), argininosuccinate synthetase 1 (ASS1), and arginase 1 (ARG1), enzymes of the urea cycle, exhibit circadian rhythms in the liver^16^. A nighttime increase in citrulline was observed in the present study; however, the citrulline/ornithine ratio, which is related to arginine consumption, was lower during the day and higher at night. This suggests that the urea cycle is activated during the day, whereas large amounts of active oxygen species, nitric oxide and citrulline, may be produced from arginine via nitric oxide synthase during the night. IPA analysis further revealed that the citrulline-nitric oxide cycle, the superpathway of citrulline metabolism, and the urea cycle showed significant differences between day and night. Nitric oxide levels and nitric oxide synthase activity have been reported to be elevated at night, and their role in neurotransmission, memory, and blood pressure regulation has also been suggested^22^. In the urea cycle, a molecule of urea is produced for each revolution of ornithine. Two ATP molecules are expended to form 2 ADP molecules in the process, which provides the energy to convert ammonia to carbamyl phosphate, and another ATP molecule is consumed to form an ADP in the process that converts citrulline to argininosuccinate. Thus, the nighttime decline in the urea cycle is reasonable, given that energy metabolism also declines during the night.

Given that several medaka models of lifestyle diseases have been established, including fatty liver disease^23^, and the fact that medaka is suitable for high-throughput screening, we propose medaka as a useful model for chronotherapy research. In addition to providing insight into the molecular mechanisms underlying circadian rhythm regulations in vertebrates, these studies should help to determine the time of day that is most effective for drug administration, and will exert the least side effects.

## Additional Information

**How to cite this article**: Fujisawa, K. *et al.* Circadian variations in the liver metabolites of medaka (*Oryzias latipes*). *Sci. Rep.* **6**, 20916; doi: 10.1038/srep20916 (2016).

## Figures and Tables

**Table 1:** Variations in amino acid levels in the medaka liver, expressed as the ratio of day to night levels.

Metabolite	Ratio (ZT16/ZT4)	p-value	
Cysteine (Cys)	2.9	0.032	*
Glycine (Gly)	2.2	0.038	*
Alanine (Ala)	1.5	0.161	
Glutamic acid (Glu)	1.3	0.038	*
Glutamine (Gln)	1.2	0.545	
Proline (Pro)	1.0	0.898	
Valine (Val)	1.0	0.859	
Serine (Ser)	0.7	0.027	*
Isoleucine (Ile)	0.6	0.034	*
Threonine (Thr)	0.6	0.013	*
Asparagine (Asn)	0.6	0.086	
Histidine (His)	0.6	0.002	**
Aspartic acid (Asp)	0.6	0.013	*
Leucine (Leu)	0.5	0.013	*
Lysine (Lys)	0.4	1.7E-04	***
Arginine (Arg)	0.4	0.193	
Tryptophan (Trp)	0.3	0.117	
Methionine (Met)	0.2	0.012	*
Phenylalanine (Phe)	0.2	0.059	
Tyrosine (Tyr)	0.2	0.038	*

*p < 0.05; **p < 0.001, Student’s t-test

**Table 2:** Canonical pathway analysis using ingenuity pathways analysis (IPA; Ingenuity Systems, www.ingenuity.com).

Ingenuity Canonical Pathways	−log(p-value)	Ratio (ZT16/ZT4)
Lysine Degradation V	3.8E + 00	2.8E − 01
Adenine and Adenosine Salvage III	3.8E + 00	4.0E − 01
Citrulline-Nitric Oxide Cycle	3.6E + 00	3.6E − 01
Guanine and Guanosine Salvage I	3.0E + 00	4.3E − 01
Morphine Biosynthesis	2.9E + 00	3.8E − 01
Purine Nucleotides Degradation II (Aerobic)	2.8E + 00	2.4E − 01
Salvage Pathways of Pyrimidine Ribonucleotides	2.8E + 00	2.4E − 01
Arginine Biosynthesis IV	2.7E + 00	2.2E − 01
TCA Cycle II (Eukaryotic)	2.7E + 00	2.2E − 01
Purine Nucleotides De Novo Biosynthesis II	2.7E + 00	1.7E − 01
Nicotine Degradation II	2.5E + 00	3.0E − 01
Palmitate Biosynthesis I (Animals)	2.5E + 00	3.0E − 01
Glutathione-mediated Detoxification	2.4E + 00	2.7E − 01
Lysine Degradation II	2.3E + 00	2.5E − 01
Purine Ribonucleosides Degradation to Ribose-1-phosphate	2.3E + 00	2.5E − 01
Glutathione Redox Reactions II	2.3E + 00	5.0E − 01
Superpathway of Citrulline Metabolism	2.3E + 00	1.7E − 01
Glycine Betaine Degradation	2.2E + 00	2.3E − 01
Urea Cycle	2.1E + 00	2.1E − 01
